# Hospitals grappling with nurse exodus

**DOI:** 10.1503/cmaj.1095934

**Published:** 2021-04-19

**Authors:** Catherine Varner

**Affiliations:** Toronto, Ont.

As Canada faces its third wave of the coronavirus disease 2019 (COVID-19) pandemic, experienced nurses are leaving emergency departments for less demanding positions or retiring early.

In the fourth quarter of 2020, job vacancies in Canada’s health care sector hit a record high of 100 300 — up 56.9% from the previous year. Hospitals posted the highest job vacancy rate of any sector, reporting 15 700 more vacancies than in 2019, and health workers say emergency departments and intensive care units are disproportionately affected.

The median age of emergency department nurses in Ontario’s Brant Community Healthcare System is a decade younger than it was one year ago due to recent retirements and departures for other positions, according to Tammy Coates, a nurse and the clinical manager of the department.

“I can’t hire a seasoned nurse to save my life,” said Coates. “I have just hired four nurses who have not even graduated yet. I would not have dreamt of doing that 10 years ago.”

Junior nurses are also caring for sicker patients with much more complex needs now than before the pandemic, she said.

According to Dr. Andrea Unger, chief of emergency services at the hospital, the exodus of senior nurses has noticeably affected triage, as junior nurses are not as quick to distinguish patients who need urgent attention from those who do not.

North York General Hospital in Toronto is experiencing similar challenges. According to Dr. Paul Hannam, chief of emergency medicine, the departure of many experienced nurses in the past year has had “a massive impact on our whole team.”

**Figure:**
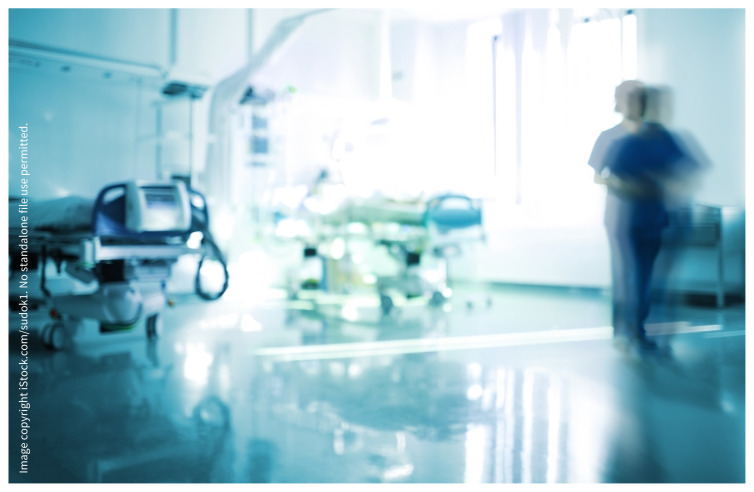
Emergency departments are struggling to retain and replace experienced nurses.

Image copyright iStock.com/sudok1. No standalone file use permitted.

“When triage doesn’t happen effectively, all of the logistical needs to care for a sick patient within the department and, to some extent, the hospital must adjust,” he said.

Nearly one in 20 nurses surveyed by the Registered Nurses’ Association of Ontario (RNAO) earlier this year said they plan to leave the profession now or immediately after the pandemic. A third of those planning to quit currently hold senior roles as nurse executives, advanced practice nurses and college faculty. Meanwhile, more than one in 10 nurses aged 26 to 35 said they were very likely to leave the profession after the pandemic — four times the normal attrition rate for that cohort.

Doris Grinspun of the RNAO said the findings of the survey were troubling. “Things have not gone smoothly in the pandemic for nurses.”

Pension plan changes may have influenced some nurses’ decisions to retire early. Initially, “many nurses who were close to retirement decided to stay to help colleagues and patients in the pandemic,” explained Grinspun. But with benefit inflation protection ending in April for those who retire early with a lump sum, “they were worried if they stayed, they would lose money for their retirement years. These changes were terribly timed.”

Heavy workloads also appear to have played a role, Grinspun said. “In intensive care units, for instance, nurses are being assigned two or three very sick patients [at a time] when, in prepandemic times, they would only have one.”

In Quebec, some 4000 nurses have quit their positions during the pandemic, up 43% over previous years. The province’s health care unions say working conditions have worsened during the pandemic due to vacation cancellations, imposed full-time work and forced overtime.

Karen Foudy, a registered nurse and executive director for Alberta Health Services, has not seen an increase in permanent departures from Calgary’s five emergency departments but noted that more seasoned nurses are requesting to move to part-time positions or take extended leaves.

“Recently, when one of our emergency nurses retired early, she said the reason was, ‘COVID just broke me,’” Foudy said. “The acuity is higher, and people have a lot going on in their personal lives.”

According to Foudy, one solution to retain nurses in acute care is to give them more support. “When people request time off, we try to give it to them because they are tired.”

“Having a model of care whereby experienced staff and redeployed staff are paired together to support one another” has also helped, she said.

In Brantford, Coates said her department has developed an extended orientation program that takes new hires through graduated front-line experiences while also pairing them with experienced nurses. One unexpected benefit of the program was that the senior nurses felt “valuable and necessary” training junior colleagues, she noted. “It seems to inject fresh, new meaning into their careers.”

